# ‘You Wouldn’t have Your Granny Using Them’: Drawing Boundaries Between Acceptable and Unacceptable Applications of Civil Drones

**DOI:** 10.1007/s11948-015-9720-7

**Published:** 2015-11-04

**Authors:** Philip Boucher

**Affiliations:** Foresight and Behavioural Insights Unit, Joint Research Centre (JRC), European Commission, 21 Rue Champ de Mars, 1050 Brussels, Belgium

**Keywords:** RPAS, UAV, Drones, Civil drones, Focus groups

## Abstract

Some industry and policy actors are concerned about public opposition to civil drones, in particular because of their association with military drones. However, very little is understood about public reactions to the technology. Strategies to ‘manage public acceptance’ have so far relied upon several untested assumptions. We conducted public engagement activities to explore citizens’ visions of civil drones. Several insights counteracted the prevailing assumptions. Rejecting the notion of blanket support for or opposition to civil drones, we found that citizens make nuanced decisions about the acceptability of civil drones depending upon the purpose of the flight and the actors involved. The results are positioned in support for calls to strengthen the role of citizens in civil drone development and, in particular, to shift away from the current focus on *citizens’ acceptance of civil drone development* towards the *development of* *civil* *drones that are acceptable to citizens*.

## Introduction

Remotely piloted aviation systems (RPAS) or, more commonly, ‘drones’ are aircraft systems that operate without an on-board pilot, either controlled by a remote pilot, an autonomous navigation system, or a combination of the two. The systems vary significantly in their size, complexity, quality and capabilities. Interest in the potential of the technology has led to calls to support the establishment of a European civil drone sector while maintaining a safe and responsible development path.

While there has been substantial debate about the ethics and societal impacts of military drones (e.g. Billitteri 2010), less attention has been paid to their civil counterparts. Many authors have highlighted issues with reference to civil liberties, particularly privacy, surveillance, and militaristic policing (Bracken-Roche et al. 2014; Finn et al. 2014; Finn and Wright 2014; Galliot 2012; Gersher 2013; Hayes et al. 2014; Jones 2014; Salter 2014; Straub 2014; Urquhart 2013). Some studies have explored the relationship between civil and military drone development (Boucher 2014b; Sparrow 2012). Several authors have called for greater and more meaningful public deliberation on how civil drones could or should be developed (e.g. Boucher 2014a; Gersher 2013; Hayes et al. 2014). Along with the development of the technology, we can also observe increasing references to civil drones in the media, particularly since 2013.^1^

The European RPAS Steering Group (ERSG) have developed strategies for managing civil drones in society, holding that it is “important to modify the vision of “killing machines” they [publics] have right now due to the actually military-specific utilisation and to some catastrophic movies” (ERSG 2013a, p30). Their proposed solution is simple; “give to the citizens a different vision” (ERSG 2013a, p36). The ERSG approach is considered representative of a sentiment often expressed in industry and policy circles where concerns are raised about potentially damaging public opposition to civil drones. Previous studies (Boucher 2014a, b) have criticised this approach for being based upon assumptions, since there have been no studies of citizens’ visions of civil drones, nor of their provenance or impact upon public acceptance. The conceptualisation of public responses to technology invoked in these strategies is also problematic, positioning the citizen as a once empty vessel that Hollywood has infected with a vision that is not compatible with acceptance. The solution is still more problematic, to cure the citizen of their opposition by simply replacing the ‘killing machine’ vision with another more compliant vision. This critique was articulated with reference to the concept of responsible research and innovation (RRI), which is described as “an inclusive approach to research and innovation (R&I)” that aims to “better align both the process and outcomes of R&I, with the values, needs and expectations of European society.” (European Commission 2014c). Specifically, three key points

1. That responsibility goes beyond the processes or outcomes of a development, extending to the substance and transparency of its purposes and motivations (Stilgoe et al. 2013).
2. That knowledge is a precondition for responsibility, so publics should become co-responsible for development, but only as agents equipped with knowledge about its motives, aims and consequences (European Commission 2013).
3. That *pushing* a technology without sufficient dialogue at early stages is irresponsible, and can damage development (von Schomberg 2013).

Considered with reference to RRI, the strategies for managing public acceptance of civil drones appear disingenuous, since the ERSG (amongst others) accept that citizens’ perspectives on technologies are important, but instead of initiating an informed debate they prefer to highlight applications with the least potential for controversy. RRI generally requires greater stakeholder involvement at earlier stages of development, and for the innovator to respond to the needs of societal actors. Boucher (2014a, b) concluded that the conceptualisation of the citizen adopted by the ERSG was unrealistic and their strategies for managing public acceptance are both unethical and unlikely to succeed. In response to these problems, he argued that the burden of acceptability should be shifted from its current focus upon the citizen, to the technology. That is, we should not focus on making citizens accept civil drones, but on making civil drones acceptable to citizens. However, doing this requires a solid understanding of citizens’ expectations and visions for the future of civil drones, an understanding we simply do not have.

The present research is positioned in response to this lack of understanding. While it seems clear that substantial public engagement work is required in the broad area of civil drone development, no studies to date have provided any deep understanding of public visions of civil drones, and so there is little work to build upon. Surveys in North America have indicated that citizens may hold sophisticated perspectives on civil drones, with support depending upon the specific use case and operator (Bracken-Roche et al. 2014; Eyerman et al. 2013). We conducted focus groups with small groups of citizens in the UK and Italy to examine the robustness of the assumptions in the strategies for managing public acceptance of drones, to explore how the participants make sense of the technology and to understand how they differentiate between acceptable and unacceptable developments. Through these sessions, we gained several interesting and, often, counterintuitive insights that may be useful in supporting ongoing developments in the field.

In the following section, we describe the study, including the aims, scope and methodological design. Following this, “Insights from the Focus Groups” section describes the insights from the focus groups, introducing several quotes, organised by theme. “Discussion” section presents a discussion of these insights before “Concluding Remarks” section offers some concluding remarks and recommendations.

## The Study

The aim of the project is to provide an early understanding of first impressions and visions of civil drones, and the boundaries of acceptability in their development. Since there was not enough existing research in the area to define which issues were important in advance, we adopted an exploratory approach in which we probe for ideas and explore them as they emerge. This contrasts with surveys which may aim to provide a representative samples of European perspectives- a task which falls outside the scope of the present research.

We defined the following research questions.

- What visions, narratives and metaphors do participants deploy to make sense of civil drone development, and where do they come from?
- What are participants’ first impressions of civil drones?
- How do citizens define the boundaries of acceptability of civil drone developments?
- How robust are the assumptions implied in the strategies for managing public acceptance of civil drones described in the ERSG (2013) roadmap?

For the remainder of this section, we describe the overall structure and methodological design of the focus groups.

To respond to our research questions while maintaining an exploratory approach, we deployed a semi-structured focus group methodology. This is because we wanted to allow the participants to define which issues were most important, and to be flexible enough to adapt the sessions around these. To do this, we worked in small groups of around eight participants with no prior knowledge of civil drones, guiding them through sets of information on the technology and asking very open-ended questions, most notably of the form ‘*what comes to mind..?*’ To do this effectively, we needed to design a script for the facilitation of sessions to help us to ensure that the discussions were productive without sacrificing flexibility. The script is reproduced in the Appendix.

Participants were not made aware that the subject was civil drones until the session had already started. This was to minimise bias, since announcing the subject may have attracted those that already held strong views, discouraged those that had little knowledge, or tempted some participants to research the subject before joining the group, perhaps affecting our ability to capture their first impressions. Since the participants were not expected to have pre-existing knowledge of civil drones, we had to provide some information for them. The information highlighted a range of potential applications without pointing directly to social or ethical problems that have been identified in the literature (such as privacy intrusion and dual-use). This maximised the opportunity for the participants to decide, through dialogue, what was important to them, and to express their reasoning in their own terms. Similarly for visions, we avoided introducing metaphors or narratives for drone development directly, anticipating that this may close down participants’ opportunities to build their own visions. This would not have been possible in a large-scale questionnaire or quantitative survey.

During the design stage, we conducted a pilot focus group with four volunteer colleagues. The purpose of this pilot was to test the structure and design of the session, to ensure the texts and videos were understandable and useful in stimulating discussion, to check that the timing was appropriate, and to offer an opportunity to consider how the method could be improved. We did not record or analyse these sessions, and do not refer to them any further here.

Following the pilot, we further developed and finalised the script and the selection of materials that we used in the session (see Appendix). The appropriate selection of materials and the design of how information is presented to the participants is crucial, because they can have a strong influence upon the resulting discussions. The task was to balance the need to give participants enough information for them to consider the technology in a useful way, without persuading them to a predefined perspective. We selected material that was more descriptive, avoiding those that are excessively normative or judgemental. The texts were taken from documents published by the European Commission and Ministry of Defence (UK), slightly modified, e.g. to remove acronyms. The images were collected from various websites, selected to illustrate the range of devices referred to as drones. This included small handheld devices, medium sized drones with different target uses, and some larger craft, comparable in size to their manned counterparts. Some of the images implied certain applications, with one including a mounted camera, and others being deployed in military, policing and industrial contexts. In order to allow the participants the opportunity to project their own ideas upon the scenario, we avoided images that referred directly to the consequences of the use either for the operator or any third parties. The videos were taken from YouTube, again, selected for their descriptive style, avoiding material that was so emotive, normative or judgemental that it could convince the participants of one perspective. For example, we referred to military use of drones without pointing to any of the more controversial aspects of their deployment. Of course, this approach has weaknesses. In particular, it may deprive participants of strong arguments in favour or opposition to civil drone development they would matter to them if they were aware of them. However, we felt that in such an early exploratory study it was more important to allow the participants a greater role in defining the problem areas. A larger follow-up study can and should give participants access to a wider range of perspectives, allowing them to develop more fully informed positions.

The final step was to organise the sessions themselves. We conducted three two-hour sessions in Manchester, UK and one in Milan, Italy, in September 2014. We contracted local companies to recruit participants, provide a venue, and to transcribe the sessions. The contractors offered small financial incentives to attend, and took responsibility for managing informed consent to participate in the research, and compliance with data protection laws. We had no direct contact with participants. Given that we were focussing on depth in small groups, there are no claims of representativeness of wider populations. Nonetheless, in order to capture a wide range of views, we did recruit a diverse range of participants in terms of age and social background, with a roughly balanced gender profile.

Each session was structured into six stages, each designed to consider specific aspects of civil drone development. The first stage was an opportunity to explore participants’ early impressions of the technology, before any substantive information was offered via the facilitator. This allowed us to learn about their immediate knowledge, assumptions and perspectives. We also designed this aspect to examine the assumption, described above, that citizens hold a *killing machines* vision of civil drones that will negatively affect their acceptance of the technology. The next three stages were based upon the three main motives for (and expected consequences of) European civil drone development (as identified in Boucher 2014b), specifically their functional benefits, economic benefits, and synergies with military drone development. A fifth stage was included to explore the video capabilities of civil drones. This was designed as an opportunity for participants to raise privacy concerns, although we did not prompt the participants by introducing privacy as a concern they should hold, preferring to allow them to articulate any such concerns in their own terms. The final stage of the sessions was dedicated to reflections. Here, we left participants to discuss together in small groups, before sharing their overall impressions and describing which questions were most important to them.

During each stage, the facilitator’s role was to maintain a useful discussion amongst the participants. At a basic level, this involved allowing discussions to develop when they were considered interesting or important and moving to the next stage when necessary. It also involved managing timekeeping and the introduction of text and video information to inform the discussions. Perhaps the most challenging aspect of maintaining a useful discussion was to maximise the opportunity for participants to develop informed perspectives and articulate them in their own terms. This required content that provided maximum information without strongly appealing to certain judgements. This was addressed during the design stage, with the selection of materials and design of the script. We avoided leading questions, such as whether participants felt that civil drone development would affect their privacy, or whether they shared the ‘killing machines’ vision of civil drones. Rather, we asked what came to mind when they considered the technology, and how they imagined the future of civil drones in society. When participants raised points directly to the facilitator they were asked to elaborate further, or the point was presented back to the group by asking whether they agreed or had a different perspective.

## Insights from the Focus Groups

In this section, we present insights from the focus groups. We took the most pertinent points from each session and grouped them together into themes. First, we present the participants’ first impressions of civil drones; their insights before receiving any detailed information about the technology and its application. This is followed by a section describing different visions articulated by participants, including metaphors and narratives used to make sense of civil drones, projections of the impacts of their development, both positive and negative, and some proposals for how development could be managed. A third section describes participants’ insights on acceptable and unacceptable use of civil drones, including how they define the distinction between the two.

Direct quotes from the focus groups are presented indented, in *italics* and are separated by paragraph breaks. Most often, the selected quotes are taken from a single statement made by one participant but, where a quote refers to an exchange between several participants, letters are used to indicate which participant is speaking. Where the facilitator of the session is cited, the quote is marked as FAC.

### First Impressions

In the first stage of the sessions we asked the participants to discuss their first impressions of civil drones. We provided a definition as ‘airborne vehicles that fly without a pilot on board, usually controlled by a pilot on the ground, that are beginning to be used for civilian purposes, including commercial, state, recreational, community and otherwise non-military uses’. Some participants had heard of the technology through applications that were reported in mainstream media, while others had no knowledge at all. However, almost all the participants had something to offer as a first impression.

Most commonly, participants referred to applications or incidents that were reported in the mainstream media. Two were particularly salient. First, a PR campaign at the peak of the pre-Christmas shopping period in 2013, including a video which demonstrated the possibility of rapid delivery of products by drones (Amazon 2014). This received significant coverage in mainstream media, and several similar campaigns followed, for example by pizza home delivery services. A second salient story came from reports of a qualifying game for the Euro 2016 football tournament, where a small drone was used to fly a contentious political flag over the pitch. The flag was retrieved and a dispute amongst players developed into violent scenes and the eventual abandonment of the game. These two incidents presented many participants’ first exposure to civil drones. From these early discussion, they appear to have had a significant impact on their first impressions of drone development.

From what I’ve seen in the news so far, they’ve been a nuisance. Like, with the football game

Without reference to specific news stories, some participants raised concerns about intrusions of privacy.

When I think of drones, I think they’re out there to watch over us.

The first thing I think of is whether our privacy is going to be invaded, a bit like ‘big brother’, really.

During these first impressions, participants in the UK sessions also expressed that their privacy had already been eroded through current surveillance practices, particularly CCTV, to the extent that further erosions would not make a substantial difference. It is also interesting to note that during this first impression session, some participants immediately accepted the inevitability of the rapid development of civil drones. This form of acceptance is better described as acquiescence, participants accepting that they have no control over the diffusion of the technology or its negative impacts.

CCTV is everywhere; you just sort of get used to it and accept it, you know, that sort of thing?

I think it’s just a matter of time before this starts coming; you know, five or ten-years. Everything’s going to be recorded. They reckon you’ve been caught on CCTV X amount of times anyway in a day so, to be honest, I don’t think it makes that much difference.

This sentiment was only expressed in the UK sessions, not the Italian ones. This may be because the ‘CCTV society’ has been an important part of public discourse in the UK for the past decades.

We did not find that the participants arrived at the session with a pre-conceived *killing machine* vision of drones as military technology. While there were isolated references to military drones, these were not framed in negative terms. Across all sessions, there were just three references to military drones during the first impressions stage. Two described military drones in neutral terms while a third referred to other people opposing them, without directly criticising military drones himself.

### Visions of Civil Drones in Society

In this section, we describe several visions (including narratives, metaphors and predictions) that the participants developed when articulating their perspective on civil drones and their development.

#### Nuisance and Criminality

Participants often discussed the potential for nuisance or criminal misuse of the technology causing trouble, such as the incident at the football game described in the previous section. In the following extract, we see a typical exchange on how the benefits of civil drones—in providing better viewing angles for football—is traded off against their vulnerability for misuse.

A: Why would you want to do that at a European football game? It’s just caused loads and loads of trouble. It just shows you though, if they cost £600 or £700, some people have daft money to spend on daft things really. I mean, who’d have thought of doing something like that? If they’re used properly though, because that was just someone larking about with it, you’d be able to get better angles for the shots and stuff, so it would enhance the football.

B: Yes, but it wasn’t done for that. It was done to have a go at each other.

A: To antagonise and scare, yes … that was just some plonker that did that at that football-, but I think they’ve got the uses.

Continuing from this theme of nuisance and misuse, participants frequently cited the possibility of using drones to enhance criminal activity. In the following examples we see reference to theft, both of commercial premises and private homes.

Criminals could easily pick up that though, couldn’t they? Like I say, for looking at banks and security guards and all sorts of things.

You just don’t want criminals using it to watch your house and things … I bet there’s people now going online and going, ‘look at them drones. We can do a job with that—look at that!’

Participants were concerned that drones could be used for stalking victims or for collecting images of children. They felt that drones offered new capabilities for such criminality and would be difficult to deal with.

Stalkers would be all over this, wouldn’t they? It would be outside her house every day, following her to the bus stop, following her to work.

We’re all decent people in the room but obviously there are plenty of people that aren’t decent people, so what they want to do with it, whether it’s to perv’ on someone or attack someone, there are plenty of reasons that I think you shouldn’t do it [allow an open market].

Participants also raised the possibility of civil drones being used by paedophiles to collect images of children without being detected.

You could have pervs watching kids. How would you ever know? You wouldn’t. Hovering above a playground at all times, if it flies over a playground.

All these peados that aren’t allowed near children’s schools will soon be able to. They’re going to love flying the bloody thing over it. [agreement]

#### Children: Deliberate and Accidental Misuse

Participants considered the combination of children and civil drones as problematic, and several participants were surprised to learn that ‘toy drones’ were widely available and within the budget of many Christmas shoppers. This scenario was expected to lead to incidents of misuse (both deliberate and accidental).

I’d be concerned about who’s controlling that. If a kid was flying that around my head and it bashed into me, I wouldn’t really want it flying around me. Who’s controlling that, to say that they can control it safely? That would be my concern.

The participants also raised that even responsible uses of civil drones by adults could be risky if used around children, who may attack them and cause accidents.

I think they’d be targets, me. Kids would be throwing stones at them if they really took off and, like you say, if they were buzzing all over the place.

#### Full Skies

Participants often adopted a vision of massive proliferation of drones, with uptake and applications extending until the norm would be to have several drones operating in the vicinity of citizens most of the time. This vision was often positioned as an inevitable consequence of opening drones to the market, and was repeatedly reinforced as a realistic vision for society in the coming years. However, while it was accepted as a plausible vision, it was usually associated with unease and was generally opposed by participants.

You’ll have to imagine them flying around the sky constantly, because if this is the mass market, that’s what there’s going to be. We’d come out of here tonight and there would be drones, drones, drones. That’s what it would be like, people delivering from one place to another, kids going around watching something as a hobby—they would just be in the sky like traffic, wouldn’t they?

#### Economy and Employment

In discussing civil drone development in the context of the economy and employment, the participants generally accepted that there would be an impact. However, most often, the expected benefits were met with some scepticism. In particular that—even if new skilled jobs are created for design, repair and piloting of civil drones—working class jobs will be cut. The Amazon video was particularly evocative in this regard, leading to concern about the distribution of benefits of civil drone development.

Well, it might create 70,000 but again, it might make how many people lose their jobs.

It’s only going to be a case of the rich are going to get richer and the poor are going to get nothing, because you put a delivery driver—who will be a normal working-class person—out of a job and then these drones are taking over.

Other participants disagreed, drawing comparison with the development of the internet to argue that society would adapt. However, the claim that civil drone development could can be compared with internet development (as presented in European Commission 2014b) were not widely accepted.

I’d perhaps be surprised with what it says here: ‘could be compared to the development of the Internet in the 90s’ [from European Commission 2014a]. That’s a strong statement because, I mean, that’s just been totally world-changing, hasn’t it? Now, would drones be world-changing?

#### Surveillance, Privacy and Security

The idea of civil drone development leading to increased surveillance and intrusions of privacy was often raised, meeting with substantial agreement that this would be a negative development. Nonetheless, many participants expressed a resignation or acquiescence to further intensive and systematic surveillance as a simple extension of CCTV technology.

We’ve been brought up with that for the last 20/30-years, we’ve always been watched.

Some participants pointed out that surveillance from civil drones is different to more traditional forms of surveillance because it may not be clear who is conducting the surveillance or why.

If, for example, it was a policeman, you could stop, have a conversation and find out. You wouldn’t know why that was following you or what’s going on. I guess it’s the not knowing that made me feel a bit uncomfortable … Who’s controlling it, what they’re controlling it for.

This vision was sometimes invoked with reference to films. Two references, in separate sessions, were made to Enemy of the State, which presents a vision of a future society under extreme surveillance. Similarly, one participant was reminded of The Truman Show, not because of the use of drones but because of the vision of a society under extreme surveillance.

It reminds me of the Truman Show. You know, the Truman Show where there are cameras on every street, where he was watched? It reminds me a bit like that.

One of the more interesting aspects of the participants’ responses to privacy aspects of civil drones was the idea that they could accept heightened surveillance, but only if they were not reminded of its presence continually.

A: The visibility of it is an issue.

FAC: Do you mean whether you can see it?

A: Yes. If you can see them there it would be a bit disconcerting, rather than if they were there and you can’t see them. I think the visibility of a drone-,

FAC: So, you’d prefer not to see it?

A: Yes, because I’d feel like it would be a matter of space; living on a spaceship or in a futuristic film or something, if there were just drones flying around.

In this sense, participants were more concerned about the feeling of having their personal space intruded than they were about excessive surveillance or privacy intrusion, which they accepted as an inevitable fact of life.

A: They’d have to be silent.

B: Yes, they would have to be silent.

FAC: Is it better if they’re silent?

A: Yes. Well, if they’re going to intrude on your privacy that much, they need to be quiet because I can’t deal with that.

#### Military Use and Terrorism

Participants were very supportive of the use of drones in military contexts and did not raise any criticisms of the relationship between the military and civil drone sectors. The main point of concern in this regard was that terrorists could also use drones.

A: There’s nothing negative you can say about that [military use of drones], nothing at all.

B: Unless the other side get it.

A: Yes, that’s true, yes.

One participant was concerned about the future developments in the international landscape. Again, the critique is not against the military drones *per se*, but about their potential use by enemies.

If the drones keep going and developing and developing and developing, Israel, for example, what if they turn on us in the future? We don’t know what’s going to happen in the future. If we’re actively increasing the capability of drones that can cause mass destruction and death, surely that’s not-, well, it’s a positive if you’re fighting a war that you want to win, but if someone turns on us-, what if America turns on us in the future? You don’t know what the future is going to entail and if we’re just pumping money into drones-

Concerns about military drones were much more often expressed with reference to the use of civil drones by terrorists. The normalisation of widespread use of drones in civil airspace was frequently identified as introducing vulnerabilities to terrorism. This vision linked strongly with the full skies vision which acts, in this case, as a master narrative.

I think it would be quite a big threat to security with terrorism and things like that, the way it is at the moment, that it can carry objects like that. What’s to say it can’t carry an explosive or something like that and drop it into a really busy area, undetected. No one would see it coming, would they, really?

The more drones that are in the sky, if they did want to attack us, we wouldn’t know it was coming because we’d just think it was an Amazon one coming over, another one.

It’s slightly unnerving, thinking of all these things flying around. Someone could just stick lasers on them and start zapping people.

One participant raised a storyline from the television series Homeland, which she said led her to worry that that civil drones may be hijacked by terrorists and used against us.

I watch a lot of crime scene stuff and, I think it was Homeland, there was one about an unmanned aircraft and someone hacked into the army’s database and took over the pilot’s unmanned aircraft. They sent the bombs, so things like that could happen; someone could hack into these unmanned aircraft and if they are equipped with rockets … then it could be a massive problem.

### Acceptable and Unacceptable Use

The visions of civil drones in society, as described in the previous section, are largely negative. However, the participants did recognise several positive points and acceptable uses of the technology. In this section, we explore how the participants defined the boundary between acceptable and unacceptable uses of civil drones, as well as their proposals for codifying these boundaries in regulation. An important point here is that the acceptability of applications often hinged upon who the user was, rather than the specifics of the use itself. Some users were trusted to ensure safety and ethical integrity while others were not trusted at all.

I think it’s disgusting. That you can just buy them. They shouldn’t be just available to the public.

I think the police force would be acceptable; I think the military is acceptable… I just feel for personal use that is not acceptable … you don’t need it, do you?

You wouldn’t have your granny using them, would you?

Applications were considered acceptable where a serious social benefit could be identified. Such serious social benefits include those that accrue to others, not just the user, and are not considered frivolous. Amongst the acceptable uses, military and police applications stand out as particularly well supported while, amongst the unacceptable uses, recreational and personal use was the most strongly opposed. The two sides of the boundary are further explored in the following two subsections before a third subsection describes the importance of avoiding function creep that could violate the boundaries of acceptability and a final subsection outlines some regulatory solutions identified by the participants.

#### Approval of Applications with Serious Social Benefit, Especially Police and Military Use

Participants strongly supported the use of drones by military, police and other authorities such as border patrol and emergency response.

A: Yes. I think it would be safer in Afghanistan or somewhere like that. you can send them out, check it’s safe and then send the troops out there, and you can see what’s going on before you actually send the troops in. The police as well, if they’re doing a raid or whatever, they can see if it’s safe and then the police can follow on.

B: Well, I think wherever it can save life and, you know, prevent death, then basically I think it’s a great thing. I mean, spying I’m not sure on, but anything certainly on saving life I think is great.

A: Even if it’s not saving a life. If it’s property or something of value.

Some users anticipated everyday benefits in a range of application areas of varying specificity and likelihood. While participants identified problems more often than benefits, it was clear that they maintained sophisticated positions, differentiating between applications that were clearly acceptable and clearly unacceptable.

A: I know everyone disagrees, but I’m excited to see how they will benefit society.

B: Yes, it’s going to be interesting. Just the last one with the sport [filming sport events], I was thinking, ‘yes, that would be really good.’ It will happen, so I’ll just embrace it.

A: I think they’ll be good for crime prevention and stuff like that.

B: Yes, I think it’s pretty intriguing really, the benefits that it could have in a personal and a commercial aspect

It’s a good idea when you’re say anything like drastic disasters, like when China had floods or tsunamis and you get places like Arizona that have huge, huge bushfires, and Australia and things, so it would work for that and it would be a very good idea for things like that.

A: To use them with military and everything else, great. Not for your normal walking around the street, I’m not interested.

B: Not for me. I don’t think normal everyday people should use them.

A: Companies, it depends what they are. Police, yes, that’s fine, it’s protecting you, but other than that, no.

I can see the advantages of them. I can see how, like a home security system, a drone could be used for a home security system. I know we were laughing before but I could see it helping somebody who is visually impaired.

Participants’ approval often stemmed from trust in the user and the level of control and regulation that they felt would protect society from unacceptable uses.

I don’t have an issue with regulated bodies using them, like police, councils and stuff like that, I have no issue with that whatsoever. The issue I have is, sort of, the unregulated, private use, is why I have issues. Companies, people at home buying them and using them for things. Police, councils, stuff like that will be regulated—probably by the EU and stuff like that—by international laws. I don’t have an issue with it at all.

#### Opposition to Flippant Applications, especially Recreational Use

Across the focus groups, participants frequently contrasted acceptable uses with those that had frivolous advantages that accrued only for the user. Such frivolous uses are exemplified in recreational use. Many participants also saw delivery services as offering insignificant advantages to society and, therefore, as unacceptable.

When it comes to police, search and rescue and military purposes, like intel gathering, I think that’s the benefit for it. I think that’s where it should stay, personally. Kids using drones and Amazon, just blows my head off.

I’m not that keen on it at all. I think it’s good for military and good for what’s needed, like the oilrigs, but not for general use.

I think it really all just comes down to what it is being used for. I think, ‘who is this being used by? And then, ‘what is it being used for?’ If it is being used by the police then as long as it’s not monitoring every single house in the street, if there’s one specific place that needs to be watched and if it’s military, again, instead of sending people in, like the remote bomb detonators that have been around for a long time, something like that, to help people. If it’s just, like you said, somebody’s just got it and they’re using it for whatever they want, like it says on the other side ‘hobbies’, that is probably the only one there that really worries me.

I don’t like the sound of them at all. I don’t mind them for things like disasters or floods or for checking pipes out, but just to have them for stuff to be delivered or watching you, like you say, it’s your privacy being taken away.

The strongest opposition was directed towards public use for hobby or recreational purposes without clearly defined and socially beneficial objectives.

Just the hobby one stands out the most … All the rest seem to have a purpose

It’s just the fun bit I don’t get. I don’t understand why you’d want one for fun

A: There’s a guy at work that’s got one. He’s spent about £1,000 on one.

B: What’s he bought that for, then?

A: I think he might be a pervert. [Laughter].

#### Function Creep

The participants also articulated concern about function creep, that civil drones may be introduced on the basis of applications with far reaching social benefits, before being extended beyond this mandate for frivolous applications that were not originally envisaged and are not considered acceptable. This concern was often linked to the full skies vision of drones, expressed with reference to exponential growth.

There might have been 100 websites at first, the big companies, now everyone’s got a website. Do you regulate how many there are? Who can apply for one? They would just be whizzing around all day.

How can we be sure that they’re not going to change the rules and regulations once it’s up and running? Like, to start off, it’s quite tight, the rules, and then it becomes lax.

One participant was concerned about the role civil drones could play in the emergence of a police state, whereby the authorities begin by using the technology for serious social benefits, but gradually extending their role towards mass surveillance.

Once the police get them then obviously they’re going to use them for everything they possibly want to use them for, and as the first stages come in and you let them use them, within two or three years, they’ll then be using them for close-up viewing and closer looking and you’ll then have all the experts that have got the techniques to do this with them and do that with them. It’s just like everything that’s provided to us, they show the end of the stick here, which is to say how great they are, but then you always here new stories like, you know, with the cameras in the street. They’re spying on people who are putting rubbish in the wrong bins and then suing them for it or whatever. To me, it’s great and people should know when they’re used for good things; I mean, if you’re going to use them for this, that and the other, that’s great, but once the police get them […] once the police get them, it’s then being used for one main purpose, which is to control the masses.

#### Solutions: Registration, Licensing and Insurance

Participants suggested how regulations could protect society from unacceptable uses of civil drones.

A: I think it should be controlled. I don’t think it should just suddenly become a great big mass where, just because you want it, you can have it.

B: The Internet wasn’t controlled, was it?

A: Well, maybe it should have been.

Usually, the proposals were analogous to car regulations, with mandatory licensing, registration of devices, and mandatory third party insurance.

Presumably, they’d all have to be registered at the very least, you know?

There should be something that you have to sign before you-, like, even if it’s just an e-agreement on the computer or something, and you’ve signed your name to that, you know, ‘I agree to use it properly and not fly it over playgrounds’ or whatever and then you get it delivered to you, no matter how you purchase it, as long as you’ve then signed an agreement.

Some participants saw these regulations as a means of restricting uses to those that are considered acceptable. Several participants were strongly opposed to civil drones’ open availability

I think you should have a licence for them and that licence is for a specific purpose.

I don’t think it should be available for the public to just buy them.

The following quote is repeated from the previous subsection on defining the boundaries of acceptable use, but also illustrates how closely the acceptability of applications are tied to the level of regulation the user is subjected to.

I don’t have an issue with regulated bodies using them, like police, councils and stuff like that, I have no issue with that whatsoever. The issue I have is, sort of, the unregulated, private use, is why I have issues. Companies, people at home buying them and using them for things. Police, councils, stuff like that will be regulated—probably by the EU and stuff like that—by international laws. I don’t have an issue with it at all.

## Discussion

The insights from the focus groups raised several interesting points that are relevant to current initiatives to support civil drone development in Europe. We consider the most pertinent of these in more detail in the following subsections. First, we explore again some of the most salient visions of civil drones in society—surveillance and privacy, employment and economic development, killing machines and full skies—before revisiting the boundary between acceptable and unacceptable use.

### Visions of Civil Drones in Society

#### Killing Machines

The killing machines vision is important because it is a central assumption at the heart of the ERSG strategy for managing public acceptance of civil drones. That is, that citizens hold a killing machines vision because of military use of drones and media representation. We found little evidence in support of the assumption. The vision of drones as killing machines was seldom adopted, even when discussing drones in a military context. In these cases, the vision of military use of drones was very positive, considered as a lifesaving technology just as can be expected for search and rescue operations. Indeed, the use of civil drones by hobbyists and recreational users appears to be much more damaging ‘P.R.’ than military applications. The full skies vision of ubiquitous drones (discussed in the following subsection) was much more common and salient than that of the killing machine, and first impressions were more often related to surveillance and nuisance (discussed in the subsequent subsection).

The finding that support for military drones notably higher than support for publically available drones and personal or recreational applications was surprising to the facilitator. It may be that the selection of text and video for this fourth stage of the sessions was too narrow to support a deeper debate of military drone use. The selection was designed to underline the dual use aspect of drone development without persuading the participants that this was a good or bad thing. In seeking to avoid influencing the participants’ judgements, we did not refer to the full range of arguments about the ethics of combat drones, nor to the controversies that surround their use. A larger study with lengthier sessions dedicated to dual use could explore these questions in depth. Despite this consideration, it was clear that the participants in the sessions did not come to the focus groups with a killing machines vision.

One problem that participants identified in the context of military use of drones was the potential for enemies, including terrorists and current allies that may become enemies in future, to use them against us. This aspect partially supported the assumption in the ERSG roadmap inasmuch as one participant referred to media representations as formative in this vision, specifically to the television series Homeland, which led her to worry that terrorists could take command of drones and use them in attacks. While several references were made to television and cinema, the participants’ first impressions appeared to be more strongly influenced by direct experiences or news reportage on incidents involving civil drones.

#### Full Skies

The most widely adopted vision of civil drone development was one of proliferation to the point where they are a near constant presence in our lives. In this vision, the force of numbers necessitates the establishment of ‘sky lanes’ and rules corresponding to those currently imposed on car drivers. Drones would be used for several applications, including delivery services, and interactions and encounters with them would be common. While participants occasionally met this vision with excitement, it was most often seen in negative terms, with concerns about accidental collisions, the invasion of personal space and vulnerability to deliberate criminality and malice. This is strongly related to participants’ more concrete concerns about function creep, whereby drones would be introduced for specific and exceptional purposes that are widely supported (such as emergency response or filming sporting events) before gradually developing until they are used for many everyday mundane tasks that are seen as frivolous (such as delivery services and routine police patrols). This feeling was encouraged by comparisons with vastly successful and well proliferated technologies such as the Internet and iPod, as presented to the group (via European Commission 2012, 2014b). The aim of these comparisons in the original text was to propose economic and employment benefits of growth. However, while the participants readily adopted the vision of rapid growth in daily use, they met the vision of economic and employment benefits with scepticism. This had the combined effect of participants feeling that they had no control over a rapid development that would inevitably occur and with benefits distributed unevenly across society.

This full skies vision often acted as a master vision, with others such as surveillance, criminality, terrorism and nuisance aligned with it.

#### Surveillance and Privacy

Many participants felt that intensive surveillance, particularly by video camera and online tracking, was an inevitable fact of modern life. While some felt uncomfortable with it as an intrusion of privacy, these feelings were often balanced against the increased security offered, for example through police access to CCTV footage. A curious insight from the focus groups was that participants said that they would prefer to remain unaware that they are under surveillance. They felt that they had lost their privacy long ago, but the reminder of ongoing surveillance would be more distracting or invasive than the privacy intrusion itself. The participants’ vision civil drones in society involved more surveillance and less privacy. However, the problem with this vision was not the invasion of privacy *per se*, but the more salient and visible reminder of ongoing surveillance.

This is particularly interesting in the context of responses to privacy concerns, which must balance legal definitions of intrusions of privacy against how such intrusions are experienced personally. Some have suggested as a response to potential privacy intrusion that drones that are filming should be required to emit warning sounds and lights, this making citizens more attentive to potential intrusions and offering a means of responding. However, they would also, by definition, increase the extent to which citizens *experience* intrusions of their privacy and personal space. Even if the level of intrusion is the same, the negative feeling of being subjected to surveillance may be exacerbated. This approach in turn influenced participants’ suggestions for solutions- instead of demanding notification of potential privacy intrusions, they prefer to restrict surveillance capacity entirely. For example, placing limits on the recording of video streams (e.g. so they can be used live, for navigation purposes, but not saved for subsequent viewing). It was also important to participants that pilots are registered and properly licenced and that their operations are authorised only for applications with clear social benefits, excluding filming for recreational or frivolous commercial purposes.

#### Employment and Economic Development

The participants articulated a sceptical vision the impact of civil drone development upon the economy and employment. While many accepted that the development of a European civil drone sector would create jobs for operators, manufacturers and repair work, they expected job losses elsewhere, particularly in the traditionally working-class sectors such as factories and deliveries. They often used comparisons with internet technologies to justify this expectation of unequal distribution of benefits.

### Defining the Boundaries of Acceptable Use

The boundary between acceptable and unacceptable use of civil drones appears to be most clearly defined where participants see a serious social benefit. This was often closely linked to their perceptions of the legitimacy of the user, as much as the application itself. Overall, participants approved of the use of drones by authorities, particularly the military, police and emergency services. While some participants made isolated references to police corruption and referred to others having doubts about military drone use (not to doubts of their own), these were not accepted by the groups as a whole. The response to commercial applications was mixed, with acceptability generally conditional upon creating a safer and more secure society combined with regulation to counteract deliberate and accidental misuse (such as licensing of pilots, registration of devices, and liability for operators). Uses where participants did not see a serious social benefit were generally deemed unacceptable. This included applications where the benefits are considered frivolous and insignificant, or accruing only to the user. Several commercial applications were considered frivolous, including parcel delivery and wedding photography, and recreational use was widely criticised by the participants. The unregulated availability of devices to all citizens was completely unacceptable to many, a sentiment well captured by the title quote of the present article.

Concerns about function creep were often raised along with expressions of inevitability and lack of public agency in development. Participants did not feel that they had any control over how the technology would develop. They adopted a position more akin to resignation, acquiescence or submission than acceptance. This was expressed in occasional comparisons of citizens with hospital patients or prison inmates, and the capacity to adapt to new problems and nuisances. Occasionally, this inevitability of development was expressed in a more positive form, as participants referred to how they became accustomed to other technologies such as smartphones without substantial difficulties.

The participants opposed developments in civil drones that could lead to increased vulnerability to misuse. Examples of misuse varied, covering a wide spectrum from children (accidentally or mischievously causing incidents) through criminals (particularly thieves and perverts) using drones to support their activities, to terrorists executing with drones disguised as serving civil purposes. This concern was strongly linked to the full skies vision, as the more civil drones were deployed the greater the vulnerability to misuse of all kinds. These problems were much more vivid and salient to the participants than privacy intrusion or associations with military applications.

In response to these divisions of acceptable and unacceptable applications of civil drones, the participants proposed solutions in the form of appropriate regulation. These closely followed the existing regulation of driving and car ownership, including licensing for pilots, individual registration of devices, and mandatory insurance to meet liabilities. Further, some participants argued that permits should only be granted to responsible actors for specific operations. For example, police and emergency services and some commercial operators may be granted permission to operate a drone in a certain set of specified circumstances, but not in others, and citizens should not be able to acquire and operate them freely without justification.

## Concluding Remarks

The insights presented in the previous sections may not provide a broad representative perspective of all citizens, but they do help us to understand more about how some citizens react to and make sense of civil drones. The discussions were rich and open, and the participants raised many interesting points, some of which were contrary to the dominant assumptions (e.g. the prevalence of the killing machines vision) and the expectations of the facilitator (e.g. the wide support for military drones and opposition to recreational drones). Other points were aligned with some concerns already identified in the literature (e.g. citizens’ lack of influence in shaping development). We feel that these are signs of success as, in each case, the participants identified the points that were important to them and articulated them in their own terms. Two of the participants contacted the facilitator to ask for further information on the project, which we suggest as evidence that they were well engaged by the process. Nonetheless, further studies will be required in order to verify and validate our results. We would also suggest further research to explore specific topics in detail. For example, to fully consider perspectives on the relationship between civil and military drones, sessions should use a wider range of materials, including the strong and often emotional appeals for and against military drones. While the videos and pictures used in the sessions we conducted were useful in facilitating the discussion, we feel that immersive activities—with live demonstrations of civil drones in action—may help particpants to develop and articulate which uses are acceptable to them and why.

From the sessions, it is clear that citizens can hold sophisticated perspectives on the technology, with the acceptability of use depending on several contextual factors. Certainly, the idea of outright support for or rejection of drones appears to be an inadequate means of capturing the acceptability of their development. This is consistent with the finding of a survey study undertaken in North America and further supports their conclusion that a public mandate for one set of civil drone applications cannot necessarily be used to justify others (Bracken-Roche et al. 2014). An acceptable development path should include safeguards against function creep, particularly in applications close to the boundary of acceptability.

To conclude, the aim of the study was to explore first impressions and visions of civil drones, to identify the boundaries of acceptable use, and to examine the robustness of the assumptions embedded in the strategies for managing public acceptance in the ERSG (2013) roadmap. In the following points, we summarise the insights that responded to this aim.

- The participants occasionally referred to cinema and television representations of drones, but their first impressions were mostly shaped by direct experience or incidents that were reported in the press.
- The participants’ first impressions of civil drones usually referred to nuisance and surveillance, and seldom to military drones.
- The killing machine vision of civil drones does not adequately capture the participants’ first impressions or later perspectives. In fact, military use of drones was among the most widely accepted application areas.
- Participants are wary of function creep, with civil drones introduced for clearly beneficial applications before being used for increasingly flippant purposes to the point where they are a pervasive feature of everyday life.
- A full skies vision was prominent and regularly combined with other concerns about function creep, nuisance, criminality and terrorism.
- The boundary between acceptable and unacceptable civil drone development was defined by how serious the benefits are, and who the user was. Those applications with safety and security benefits for others were considered acceptable while those with limited, frivolous benefits accruing only to the operator were not. Recreational and hobby use were the most strongly opposed application area.
- The participants often offered solutions to the problems they identified. These were usually akin to those that apply to motoring (licensing of pilots, registration of devices, mandatory liability insurance) but included others which would restrict use to trusted authorities and require authorisation, which would depend upon the application and its social benefits.
- Participants were generally resigned to the emergence and rapid development of civil drone technology and did not feel that they had any role in shaping development.
- Many participants felt that civil drone development represented a threat to their privacy. However, while such intrusions were considered a regular feature of modern life, participants were concerned about their experience of personal space, which they felt would be threatened by regular reminders of surveillance.

We believe that these insights provide an early warning for those perusing strategies to encourage citizens to accept civil drone developments, particularly from a starting point of unfounded assumptions about citizens’ perspectives and dubious conceptualisations of technology in society. In conclusion, we reiterate the call to shift from the current focus on public acceptance of civil drone development to the development of civil drones that are acceptable to society.

## Figures and Tables

**Fig. 1:**
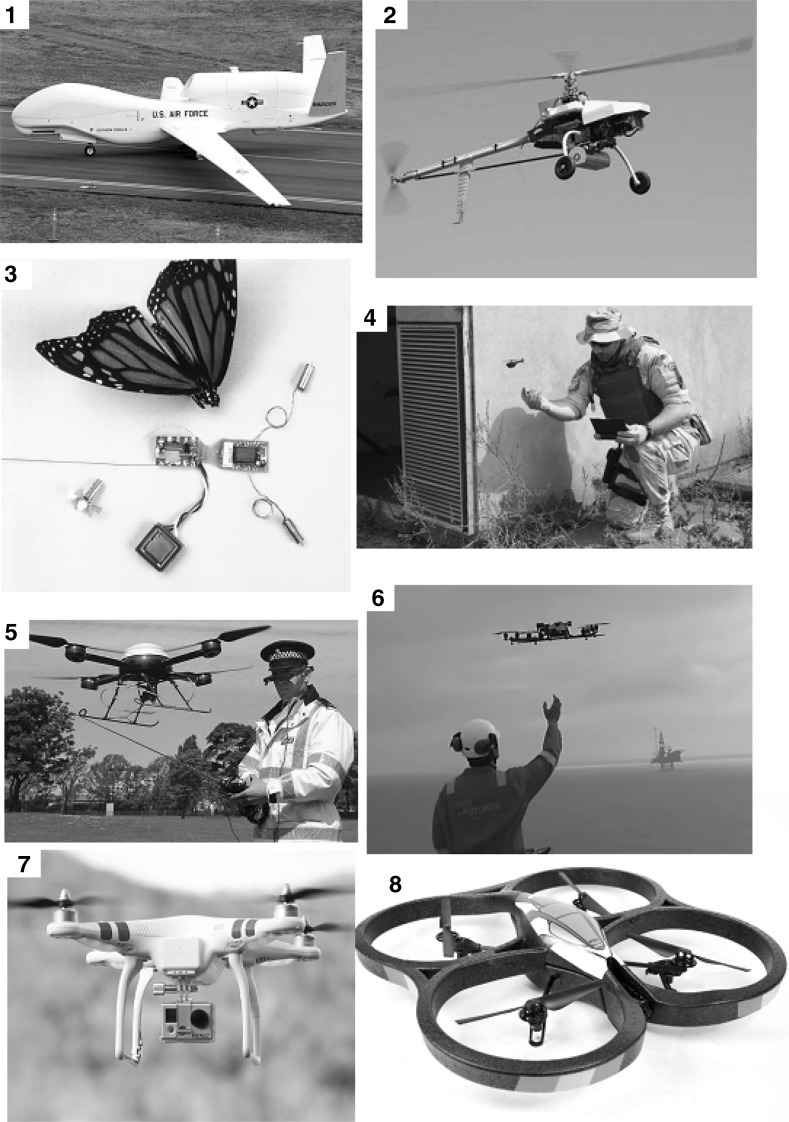
Pictures used in Focus Groups. *1* **–** *4* UVS International; *5*. John Giles/PA Wire; *6*. Offshore Energy Today; *7*. flying-drones.expert; *8*. bit-tech

## Appendix: Script and Materials

Introduce facilitator and start the session with the following information: Today, the technology we are going to talk about is civil drones. Drones are airborne vehicles that fly without a pilot on board, usually controlled by a pilot on the ground. They are beginning to be used for civilian purposes, so commercial, state, recreational, community and otherwise non-military uses. Before starting the discussion, I will explain a little more about the research.
As well as drones, my colleagues are doing focus groups on the latest internet technologies, and wearable sensors. The aim of these studies is to learn more about new technologies in society. In particular, we research questions of responsibility and ethics. We do not do market research, and we are not here to find out how to convince people to support a technology, but to learn how to make a technology that people will support. The research is used to help support policy development. You can request to be kept up to date with the latest policy developments, with the research, or to be provided with a copy of the final report [distribute contact details].
During the session, my job is as facilitator. This means I mostly try to guide a discussion between you, and provide you with information. I would like you to discuss points amongst yourselves, responding directly to others, but you may ask me any questions at any time. To help the discussion, I have some text and videos that we can read and watch together. These cover some of the main motivations for developing civil drones and some possible scenarios of their use. Moving in a circle, ask people to introduce themselves, first names only, and to share with the group anything that comes to mind when they think about drones. Hand out the pictures [reproduced in Fig. 1, below] and explain that drones are airborne vehicles that fly without a pilot on board, usually controlled by a pilot on the ground, and that they are beginning to be used for civilian purposes, including commercial, state, recreational, community and otherwise non-military uses. Referring to the pictures, show that there are many different kinds of drones, taking a range of forms and used in different ways, as implied by the photos.

After around 20 min, introduce the next stage of the session, focussing upon potential functional applications of civil drones. Tell the participants that one of the motivations for developing civil drones is that they can perform tasks that are difficult for humans, often referred to as ‘dull dirty and dangerous’ tasks. Responses to these tasks may be better served without a human presence either because of their repetitive nature or because of the risks they present. Civil drones might be useful in some such tasks, such as inspecting pipes or industrial infrastructure, or investigating incidents such as damaged buildings or lost persons. Start discussion by asking the participants if anything comes to mind, and encourage discussion amongst participants.

Show the participants two videos, showing the potential use of a drone to support fire services and to deliver parcels respectively.
http://www.youtube.com/watch?v=m7XXt3pfg [show from 1m05s to 1m50s]

http://www.youtube.com/watch?v=Le46ERPMlWU [show in entirety] Give the participants a sheet of paper with the following text excerpts to read. Civil drones are already being used for civil purposes and are expected to increasingly influence our daily lives. Just as the internet technology in the early nineties gave rise to many different applications, civil drone technologies should lead in the coming years to the development of a wide variety of different services, especially if combined with other technologies. On other continents, civil drone operators support precision farming through more effective and timely application of fertilizers or pesticides. In Europe, civil drone are being used for safety inspections of infrastructure, such as rail tracks, dams, dykes or power grids. National authorities are using them in disaster relief, e.g. to overfly flooded areas or to support fire fighting. In future, civil drone could make it possible to bring giant wind turbines into the air and produce “green” electricity. On the other end of the scale, engineers are working on micro civil drone which could be used to tackle gas or chemical leaks, or which could be programmed to act like bees to pollinate plants. (Adapted from European Commission 2014a)
I’m not sure what sort of image comes to your mind when I say that word, but here’s one example. Civil drones can inspect the underside of an oil rig—100 s of kilometres off shore. A very dangerous job for a human being. Civil drones can also check for damage on road and rail bridges, monitor natural disasters such as flooding and spray crops with pinpoint accuracy. In the future, they may even deliver books from your favourite online retailer. (Adapted from European Commission 2014c) Promote discussion by asking what comes to mind, and whether this is how the participants imagine the future of civil drone development.

After around 20 min, move to the next part of the discussion, focussing upon potential economic and employment impacts of civil drone development. Introduce one of the motives for civil drone development as the potential benefits for the economy and employment. Present the participants with a sheet of text featuring the following texts to read. It is expected that once the barriers limiting civil drone flight will be removed the understanding of the civil drone potential will quickly spread amongst potential users creating new markets of aerial services, in the same way that iPad created an entirely new and unpredicted market for mobile data services. (Adapted from European Commission 2012)
The impact of drones and their many applications on the economy could potentially be compared to the development of the internet in the nineties (Adapted from European Commission 2014b)
Mastering drone technology will become a key to the future competitiveness of the European aeronautics industry. Currently, the USA and Israel dominate global manufacturing, building on expertise in the field of large military drones. The growing drone activities will translate into a substantial number of new jobs. A US industry study forecasts that in the first three years of integration in the national airspace, more than 70,000 jobs will be created with an economic impact of more than $13.6 billion. The number of jobs created through new drone activities in the US is estimated to exceed 100,000 by 2025. For Europe, about 150,000 jobs by 2050 are forecast, plus employment generated through operator services. (Adapted from European Commission 2014a) Promote discussion by asking what comes to mind when they read these texts. Ask if they imagine civil drone development will bring these economic and employment benefits, and if they believe it is important for Europe to compete with drone producers elsewhere in the world.

After around 20 min, approximately 1 h will have passed. Where appropriate, break for 5 min.

Introduce the next part of the session, focussing upon the relationship between civil and military drones. Tell the participants that, as well as the potential economic and functional benefits already discussed, one of the motivations for civil drone development is to support the military drone sector. Do not raise critique of this relationship, or of military use of drones, but allow them to discuss it if they wish. Present the participants with a sheet of text featuring the following texts to read. The changes in world economies over the last 2 decades mean that the military sector is now dwarfed by the economic size and power of the commercial sector. Except perhaps for space, new developments in military systems are therefore likely to come from specialised development of commercial systems rather than vice versa. It is to the commercial sector that we must look for the delivery of future disruptive technology. (Adapted from Ministry of Defence 2011) Show the participants the following video, showing how a very small device that was developed for civilian purposes (search and rescue) is used by the UK military in Afghanistan.
http://www.youtube.com/watch?v=1tetyswGyGA [show in entirety] Promote discussion by asking what comes to mind, whether they believe it matters that civil and military drone development are linked.

Introduce the next part of the session, focussing upon camera capabilities of drones. Explain that most applications involve the use of cameras for photo and, more frequently, video capture. Give examples of recreational photography, professional video recording, and police and border surveillance. Do not mention privacy concerns, but allow participants to discuss the use of cameras in their own terms. Show the participants the following video, showing footage recorded from a drone, and leave it on during the discussion.
http://www.youtube.com/watch?v=IBgwslpsHoc [show during discussion] Promote discussion by asking what comes to mind. If the discussion does not develop, ask if it matters to them that the footage included people’s houses.

After around 20 min, introduce the final stage of the session, devoted to reflections. Split the participants into groups of three, and ask them to discuss together for 5–10 min (depending on time remaining in the session) their overall reflections of civil drones and their development. Do not get involved in these discussions, but remain available for those that have questions. Give each group a sheet of paper with the following instructions: Reflect in your groups on the civil drones. Note any key points you would like to make. Imagine that you are meeting a group of the most important experts in charge of the development of civil drones. The group includes:

- Policymakers in charge of civil drone development
- Key companies and industry representatives
- Scientists and technology experts

You can ask questions before you will have the chance to decide on the rules for civil drones. What are your questions? Talk together in your group and decide amongst yourselves, before writing them below. Following this period of reflection, invite the participants to share their reflections and key questions with the group. Allow discussion to develop when other participants wish to respond (time permitting).

As the session draws to a close, invite the participants to get in touch if they have any questions or would like to be kept up to date with the project.

## Footnotes

1 A quick survey of the UK and Italian media reveals very rare references to civil and non-military drones (1–2 articles per year) until the period starting September 2011. For the next two years, limited reportage is identified (1–3 articles per month) before a drastic increase of around 300 % during the 12 months immediately preceding the present research (i.e. from September 2013).
